# Systematic antibody generation and validation via tissue microarray technology leading to identification of a novel protein prognostic panel in breast cancer

**DOI:** 10.1186/1471-2407-13-175

**Published:** 2013-04-02

**Authors:** Patrick C O´Leary, Sarah A Penny, Roisin T Dolan, Catherine M Kelly, Stephen F Madden, Elton Rexhepaj, Donal J Brennan, Amanda H McCann, Fredrik Pontén, Mathias Uhlén, Radoslaw Zagozdzon, Michael J Duffy, Malcolm R Kell, Karin Jirström, William M Gallagher

**Affiliations:** 1 UCD School of Biomolecular and Biomedical Science, UCD Conway Institute of Biomolecular and Biomedical Science, University College Dublin, Dublin 4, Ireland; 2 UCD School of Medicine and Medical Science, University College Dublin, Dublin 4, Ireland; 3 Molecular Therapeutics for Cancer Ireland, National Institute for Cellular Biotechnology, Dublin City University, Glasnevin, Dublin, Ireland; 4 Department of Pathology, University Hospital of Uppsala, Uppsala, Sweden; 5 Royal Institute of Technology, Stockholm, Sweden; 6 Department of Breast & Endocrine Surgery, Mater Misericordiae University Hospital, Dublin 7, Ireland; 7 UCD Clinical Research Centre, St. Vincent’s University Hospital, Dublin 4, Ireland; 8 Division of Pathology, Department of Clinical Sciences, Lund University, Skåne University Hospital, Lund, Sweden

**Keywords:** Prognostic biomarkers, Tissue microarray, Breast cancer, Antibody screening, Antibody validation

## Abstract

**Background:**

Although omic-based discovery approaches can provide powerful tools for biomarker identification, several reservations have been raised regarding the clinical applicability of gene expression studies, such as their prohibitive cost. However, the limited availability of antibodies is a key barrier to the development of a lower cost alternative, namely a discrete collection of immunohistochemistry (IHC)-based biomarkers. The aim of this study was to use a systematic approach to generate and screen affinity-purified, mono-specific antibodies targeting progression-related biomarkers, with a view towards developing a clinically applicable IHC-based prognostic biomarker panel for breast cancer.

**Methods:**

We examined both in-house and publicly available breast cancer DNA microarray datasets relating to invasion and metastasis, thus identifying a cohort of candidate progression-associated biomarkers. Of these, 18 antibodies were released for extended analysis. Validated antibodies were screened against a tissue microarray (TMA) constructed from a cohort of consecutive breast cancer cases (n = 512) to test the immunohistochemical surrogate signature.

**Results:**

Antibody screening revealed 3 candidate prognostic markers: the cell cycle regulator, Anillin (ANLN); the mitogen-activated protein kinase, PDZ-Binding Kinase (PBK); and the estrogen response gene, PDZ-Domain Containing 1 (PDZK1). Increased expression of ANLN and PBK was associated with poor prognosis, whilst increased expression of PDZK1 was associated with good prognosis. A 3-marker signature comprised of high PBK, high ANLN and low PDZK1 expression was associated with decreased recurrence-free survival (*p* < 0.001) and breast cancer-specific survival (BCSS) (*p* < 0.001). This novel signature was associated with high tumour grade (p < 0.001), positive nodal status (p = 0.029), ER-negativity (p = 0.006), Her2-positivity (p = 0.036) and high Ki67 status (p < 0.001). However, multivariate Cox regression demonstrated that the signature was not a significant predictor of BCSS (HR = 6.38; 95% CI = 0.79-51.26, *p* = 0.082).

**Conclusions:**

We have developed a comprehensive biomarker pathway that extends from discovery through to validation on a TMA platform. This proof-of-concept study has resulted in the identification of a novel 3-protein prognostic panel. Additional biochemical markers, interrogated using this high-throughput platform, may further augment the prognostic accuracy of this panel to a point that may allow implementation into routine clinical practice.

## Background

Breast cancer is a heterogeneous disease driven by a continuum of mutations and abnormal gene/protein expression that controls the tumourigenic phenotype and molecular mechanisms underpinning the complexity of its clinical behaviour [1]. To select systemic therapies, current treatment guidelines combine traditional prognostic factors (stage, tumour size, histologic grade, nodal status) with estrogen receptor (ER), progesterone receptor (PR) and human epidermal growth factor receptor 2 (Her2) expression status. However, these conventional prognostic algorithms are insufficient to capture the biologic diversity of breast cancer and impede effective tailoring of individualised treatment strategies [2]. In the post-genomic era, advances in prognostic and predictive models are beginning to capture this heterogeneity, not least with the recent generation of a new molecular classification consisting of at least ten different breast cancer subtypes [3-6]. Molecular profiling of cancer tissues has aided the development of targeted therapies, improved our understanding of treatment resistance, and helps better predict patient prognosis. This knowledge has allowed personalised breast cancer therapeutic regimens to become an achievable goal.

The cornerstone of molecular profiling has historically been transcriptomics which has transformed our understanding of the complexity of the underlying signalling pathways and interactions within a breast tumour, as well as allowing the identification of gene expression signatures associated with patient outcome [4,7]. Consequently, clinical development of transcriptomic profiling tools has dramatically escalated, augmenting standard diagnostic and prognostic information obtained from traditional clinicopathological variables [8]. The most clinically advanced prognostic gene expression signatures in breast cancer are MammaPrint [7,9] and OncotypeDx [10], which are currently the subject of large-scale prospective randomised control trials to assess their utility for stratification of breast cancer patients [11-13].

Whilst transcriptomic approaches have undoubtedly enabled the acceleration of translational pathology, providing an excellent platform for omic-based discovery [13,14], reservations have been raised regarding the clinical applicability of gene expression studies given their prohibitive cost, often reliance on frozen tissue, quality assurance issues and the advanced technical expertise required to utilise the technology [2]. Crucially, mRNA transcription does not necessarily translate to protein expression, and it is not uncommon to observe a discrepancy between mRNA and protein expression [15,16]. As proteins are one of the primary effectors of the cell, protein-based assays may be more clinically relevant as biomarkers in personalised medicine. Effective implementation of personalised cancer therapy depends upon the successful identification and translation of informative biomarkers to aid treatment provision. In a prior review, we described the contribution of antibody-based proteomics for fast-tracking the development of new diagnostic assays that are crucial to achieving personalisation of cancer therapy [17]. The systematic generation and validation of specific antibodies offers a high-throughput mechanism for the functional exploration of the proteome and a logical approach for fast-tracking the translation of identified biomarkers [17]. Whilst DNA microarray technology provides an excellent platform for biomarker discovery, it would now appear that IHC and genomic sequencing may play an increasingly important role in the clinical management of breast cancer [2]. Tissue microarrays (TMAs) are an ideal platform for rapid development of an IHC profile, allowing multiple targets to be systematically assessed, and reduce an assay to clinical utility [3-5,8,18-23].

In this proof-of-concept study, we used a novel high-throughput system, using affinity-purified, mono-specific antibodies, to translate protein targets from gene expression studies into clinically applicable IHC-based prognostic panels for breast cancer.

## Methods

### Selection of candidate biomarkers from transcriptomic datasets

Thirty-one genes were selected from an in-house analysis of the van ’t Veer study [7], using a Between Group Analysis (BGA) method identifying the top 100 good and poor prognosis genes [24,25]. From this list, we considered the top 15 genes associated with good prognosis and the top 16 genes associated with poor prognosis. Another 25 genes of interest were selected from a transcriptomic study of ductal carcinoma *in situ* (DCIS) to invasive ductal carcinoma (IDC) progression, with a particular focus on transcripts that were up-regulated in the invasive component [26] (Additional file 1: Table S1).

### Patients

The TMAs used in this study were derived from a reference cohort of 512 consecutive invasive breast cancer cases diagnosed at the Department of Pathology, Malmö University Hospital, Malmö, Sweden between 1988 and 1992 and have been previously described [27-29]. The median patient age was 65 years (range 27–96) and median follow-up time regarding disease-specific and overall survival was 11 years (range 0–17). Duplicate cores for each patient were reported as consensus scores. Each patient was assigned a unique identifier that was then linked to an anonymised ethics board-approved database containing follow-up information. Patients with recurrent disease and previous systemic therapies were excluded. Two hundred and sixty-three patients were deceased at the last follow-up date (December 2004), 90 of which were classified as breast cancer-specific deaths. Ethical permission was obtained from the Local Ethics Committee at Lund University (Dnr 613/02), whereby informed consent was deemed not to be required, but opting out was an option.

### TMA construction

The TMAs were constructed using a manual tissue arrayer (MTA-1, Beecher Inc., WI, USA). PBK and PDZK1 were screened on a TMA inclusive of all 512 cases from the reference cohort with 0.6 mm duplicate tissue cores extracted from each donor block. ANLN was screened on a second generation TMA inclusive of 498 cases from the reference cohort, with 1.0 mm duplicate tissue cores extracted from each donor block and transferred to the recipient block. The total number of cores per block was limited to ~ 200 (100 patients), with a total of 5 blocks arrayed.

### Antibody generation

The Human Protein Atlas (HPA) [30] use a high-throughput method to generate affinity-purified, mono-specific antibodies raised to all non-redundant human proteins [31]. Protein epitope sequence tag (PrEST)-specific antibodies represent unique regions of each protein target. Rabbit polyclonal antisera immunised with His_6_ABP-PrEST antigens derived from a subset of the 56 targets of interest described above (Additional file 1: Table S1) were purified by a two-step immunoaffinity protocol to obtain pure mono-specific antibodies [32].

### Cell culture

A panel of breast epithelial cell lines were selected to test antibody specificity, including MCF-7, BT474, T47D, SKBR3, MDA-MB-231 and Hs578T cells. The Hs578T (i8) invasive subclone was a kind gift from Dr. Susan McDonnell (School of Chemical & Bioprocess Engineering, University College Dublin, Ireland) and was derived from the parental Hs578T cell line (also denoted as Hs578T(P)) by sequential selection through the BD Matrigel® Invasion Chamber assay system [33]. All remaining cell lines were purchased from the European Collection of Cell Cultures (Wiltshire, UK). The MCF-7, BT474, T47D, SKBR3, and MDA-MB-231 cell lines were cultured in DMEM supplemented with 10% (w/v) foetal calf serum, 2 mM L-glutamine, 50 IU/ml penicillin, and 50 μg/ml streptomycin sulphate. The Hs578T variants were also supplemented with 10 μg/ml bovine insulin. Cells were maintained in humidified air with 5% CO_2_ at 37°C. Studies of protein expression were performed on cells at 70-80% confluence. All cell lines were routinely screened for Mycoplasma contamination.

### Western blot analysis

Total protein was extracted from sub-confluent cells by the addition of radioimmunoprecipitation assay buffer (RIPA), followed by centrifugation at 16,000 g for 20 min at 4°C. The supernatants were removed and the protein levels determined using the bicinchoninic acid (BCA) method (Pierce, IL). Samples containing 50 μg aliquots of protein were separated by sodium dodecyl sulfatepolyacrylamide gel electrophoresis (SDS-PAGE), on a 12% polyacrylamide gel under reducing conditions. Following electrophoresis, proteins were transferred to polyvinylidene fluoride membrane. Membranes were blocked in 5% non-fat milk for 1 hr at room temperature. Protein expression was detected using rabbit mono-specific polyclonal anti-human antibodies (HPA, Sweden) applied overnight at 4°C (PDZK1 1:1000 dilution; PBK, ANLN 1:500). Membranes were washed in TBS-T (Tris buffered saline with 0.1% Tween 20) and incubated for 1 hr with horseradish peroxidase (HRP)-conjugated anti-rabbit immunoglobulin (all antibodies: 1:5000 dilution). The blots were again washed in TBS-T. HRP was detected using Enhanced Chemiluminescence plus (Amersham Biosciences, UK). Chemiluminescence was detected by autoradiography using X-ray film. Membranes were stripped and re-probed with anti-β-actin (1:5000 dilution; Abcam, UK) as a loading control.

### Cell pellet arrays

In order to validate the Western blotting results in the IHC setting, a cell pellet array was constructed and IHC was performed on the same panel of breast cancer cell lines. Cells were trypsinised and fixed for 1 hr in 10% formalin, centrifuged at 500 x g for 10 minutes, washed twice with PBS and re-suspended in 0.8% agarose. The tumour cell-containing agarose plugs were processed through gradient concentrations of alcohols before being cleared in xylene and washed in molten paraffin. These cell pellets were embedded in paraffin and arrayed in quadruplicate 1.0 mm cores using a manual tissue arrayer (MTA-1, Beecher Inc, WI). IHC was carried out on 5 μm sections.

### Immunohistochemical analysis

Sections of cell pellet arrays or TMAs were deparaffinised in xylene and rehydrated in descending gradient alcohols. Heat-mediated antigen retrieval was performed using 10 mM sodium citrate buffer (pH 6.0) in a PT module (LabVision, UK) for 15 min at 95°C. The LabVision IHC kit (LabVision, UK) was used for staining. Endogenous peroxidase activity was blocked by incubation with 3% hydrogen peroxide for 10 min. Sections were blocked for 10 min in UV blocking agent. Rabbit polyclonal anti-human antibodies (HPA, Sweden) were applied at individual optimised dilutions for 1 hr (PDZK1 1:50 dilution; PBK, ANLN 1:150). Sections were washed in phosphate buffered saline with 0.1% Tween 20 (PBS-T). Subsequently, primary antibody enhancer was applied for 20 min, and sections were washed again in PBS-T. Sections were then incubated with HRP polymer for 15 min, washed in PBS-T and then developed for 10 min using diaminobenzidine (DAB) solution (LabVision, UK). After antigen retrieval, all incubations and washing stages were carried out at room temperature. The sections were counterstained in haematoxylin, dehydrated in alcohol and xylene and mounted using an automated coverslipper (Leica, Germany). As a negative control, the primary antibodies were substituted with PBS-T.

### Evaluation of immunohistochemical staining

Slides were scanned at 20X magnification using a ScanScope XT slide scanner (Aperio Technologies, CA). Cores with less than 30% tissue present or less than 100 cells were discarded to avoid manual selection bias. Tumour samples were evaluated by at least two independent observers including one pathologist, and the maximum values of the two cores was used. All discordant cases were re-evaluated and a consensus reached between both observers. ANLN expression, as a nuclear marker, was categorised based on percentage nuclear staining such that 0 = ≤1%, 1 = 2-25%, 2 = 26-75% and 3= > 75%. PDZK1 expression, as a cytoplasmic marker, was scored on a semi-quantitative scale depending on intensity of cytoplasmic staining: ranging from 0–3, where 0 is negative, 1 is weakly positive, 2 is medium positive and 3 is strongly positive. The intensity distribution (ID) scoring method was used with the cytoplasmic marker, PBK, which incorporated intensity of the scoring with percentage of cells stained [34].

### Annotation of gene expression data and hybridisation probes

Gene expression data sets were downloaded from the Gene Expression Omnibus [35] or authors’ websites in the form of raw data files where possible (Additional file 1: Table S2) [36-43]. Relevant gene expression and clinical data was extracted from ten publicly available datasets incorporating approximately 1,300 samples. Where raw data was not available, the normalised data as published by the original study was used. In the case of the Affymetrix datasets (.cel files), gene expression values were called using the robust multichip average method and data were quantile normalised using the Bioconductor package, affy [44,45]. For the dual-channel platforms, data were loess normalised using the Bioconductor package limma [46]. Hybridisation probes were mapped to Entrez gene IDs to gene-centre the data [47]. The Entrez gene IDs corresponding to the array probes targeting genes of interest were obtained from the Gene database at NCBI [48] (ANLN:54443, PBK:55872, PDZK1:5174). If there were multiple probes for the same gene, the probes were averaged for that gene. All calculations were carried out in the R statistical environment [49].

### Statistical analysis of transcriptomic meta-analysis data

Gene expression data from ten publicly available datasets were included in a meta-analysis to evaluate the individual prognostic significance of candidate proteins at the transcriptomic level, as previously described (Additional file 1: Table S2) [36-43]. Once a sample was assigned to a particular group, the 10 datasets were combined and a global survival analysis was performed. Each dataset was considered separately when determining which group a sample belonged to, due to the variability across different platforms. Recurrence-free survival (RFS) was considered the survival end point. Median mRNA levels established the cut-off for high and low expression for each biomarker. Survival curves of the dichotomised groups were compared using the log-rank test for significance. The survival curve was based on Kaplan-Meier estimates. Cox regression analysis was used to calculate hazard ratios (HR) and to adjust for all available clinical parameters. Across the meta-analysis, the available clinicopathological parameters were lymph node status, tumour grade and ER status.

### Statistical analysis of consecutive cohort data

The χ^2^ test and Fisher’s exact test were used to evaluate associations between protein expression and clinicopathological variables in the cohort. Pearson’s correlation coefficient was used to evaluate correlation between expression of the three independent markers. Kaplan-Meier analysis and the log-rank test were used to illustrate differences between recurrence-free survival (RFS) or breast cancer-specific survival (BCSS), according to differential protein expression. Cox proportional hazards regression was used to estimate proportional hazards for the individual protein expression and other clinicopathological variables in both univariate and multivariate models. The clinicopathological variables available for the consecutive cohort included tumour size, age at diagnosis, histological type, grade, nodal, ER, PR, Ki67 and Her2 status. All calculations were carried out using IBM SPSS Statistics version 20.0.

## Results

### High-throughput screening platform for mono-specific antibodies against candidate breast cancer progression-related biomarkers

**Figure 1:**
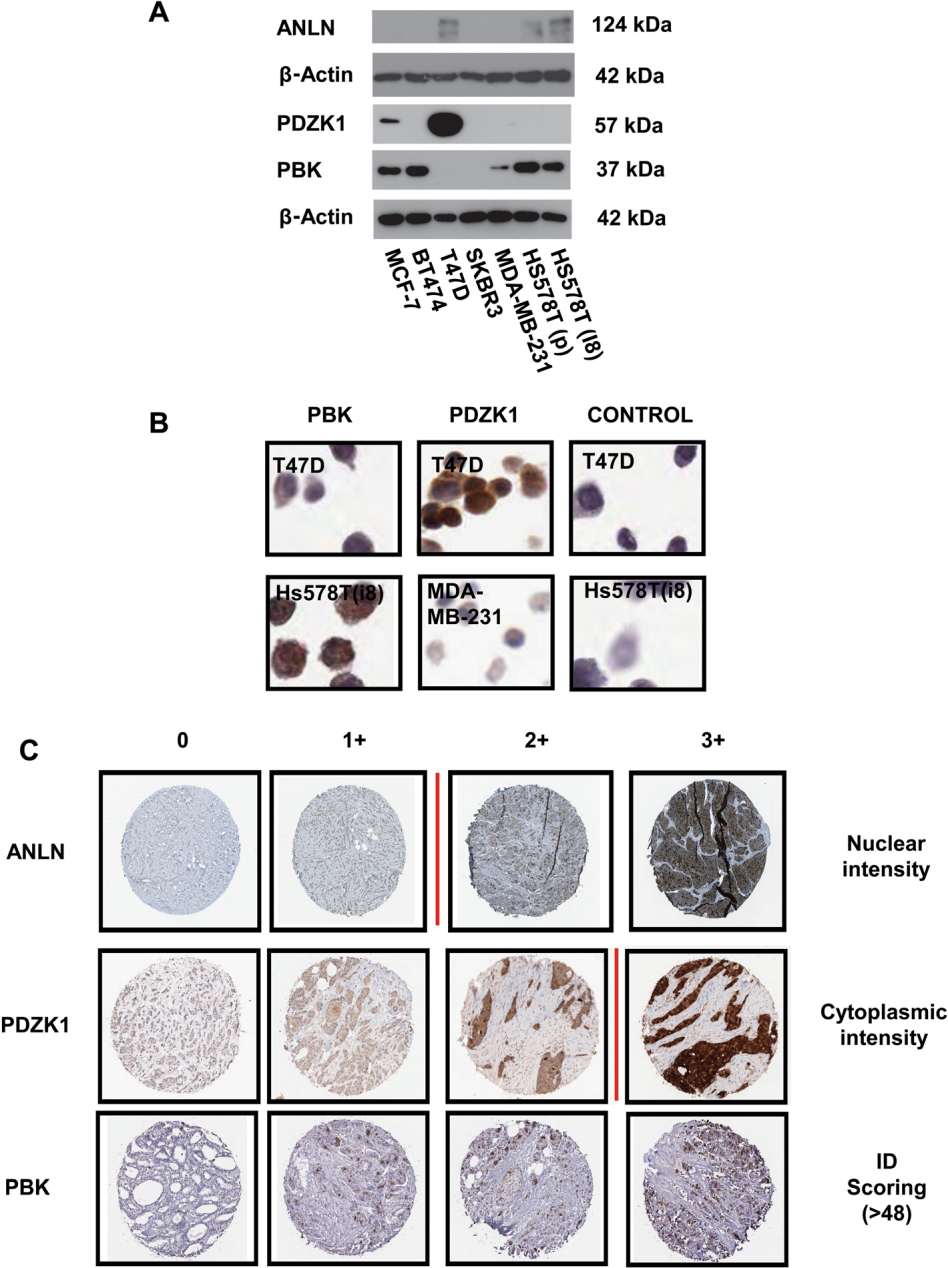
**Expression of PBK, PDZK1 and ANLN protein in breast cancer. A**: Western blot analysis of PBK, PDZK1 and ANLN protein expression across a panel of 7 breast cancer cell lines of varying invasive capabilities. ANLN antibody specificity also validated by shRNA-mediated knockdown (data not shown). **B**: Validation of the PBK and PDZK1 antibodies by immunohistochemistry in a panel of FFPE breast cancer cell lines (x20 magnification). The T47D, MDA-MB-231 and Hs578T (i8) cell lines are specifically shown. Antibody positivity is indicated by the brown DAB staining. **C**: Representative cores of ANLN, PDZK1 and PBK protein expression from the TMAs graded on a scale from 0 to 3+ for protein staining intensity. Vertical red line represents the cut-off between low and high protein expression for each biomarker.

In this study, fifty-six gene targets of interest were selected for generation of polyclonal affinity-purified anti-PrEST anti-sera on the basis of links with breast cancer progression at the mRNA level in previously published transcriptomic datasets [7,25,26]. Of the 56 gene targets submitted to the HPA, 18 mono-specific antibodies were released for extended analysis. Specificity of the 18 antibodies was initially validated by Western blot analysis on a panel of discrete breast cancer cell lines with varying invasive properties. Ten out of the 18 antibodies exhibited specificity via Western blot analysis, with the expected molecular weight being observed (ANLN, PDZK1 and PBK shown in Figure 1A). Specificity was further verified by performing IHC on the corresponding formalin-fixed, paraffin-embedded (FFPE) breast cancer cell lines (subset shown in Figure 1B). Seven antibodies showed concordant results for Western blot analysis and IHC staining in the breast cancer cell line cohort. Finally, three antibodies (PDZK1, ANLN, PBK) were successfully optimised on full-face paraffin embedded sections of breast cancer tissues and subsequently selected for screening on TMAs (Figure 1C).

### Protein expression of lead candidate biomarkers in breast tumours determined by IHC

As shown in Figure 1A, antibodies against anillin (ANLN), PDZ-Domain Containing 1 (PDZK1) and PDZ-Binding Kinase (PBK) demonstrated specificity via Western blot analysis and exhibited concordant IHC staining on cell pellet arrays across 7 breast cancer cell lines. Antibody specificity was further validated using Western blotting and antigen microarrays (Additional file 2: Figure S1).

Four hundred and seventy-nine of the original cohort of 512 tumours (93.6%) were available for analysis of ANLN immunostaining, with 280/512 (54.7%) available for analysis of PDZK1 immunostaining and 292/512 (57.0%) available for analysis PBK immunostaining, with several sets of tumours not available for analysis due to core loss. Two hundred and fifty-two out of 512 (49.2%) tumours had a score for each of the three biomarkers, while 260 were not available for analysis due to core loss in the case of at least one of the 3 markers under evaluation. The clinicopathological variables for the available (n = 252) and unavailable (n = 260) tumours were compared using χ^2^ analysis and Fisher’s Exact test, with no significant difference being seen in terms of patient age (*p* = 0.927), tumour size (*p* = 0.582), tumour grade (*p* = 0.271), histological type (*p* = 0.368), nodal status (*p* = 0.479), ER status (*p* = 0.578), PR (*p* = 0.612), Her2 (*p* = 0.192) or Ki67 (*p* = 0.754) expression between available and unavailable samples.

Using semi-quantitative analysis, IHC staining was scored on a scale of 0–3 based on intensity of staining (ANLN and PDZK1) or based on ID scoring (PBK) (see Figure 1C). High ANLN protein expression were classified as tumours with a staining intensity >1, and low expression classified as tumours with a staining intensity ≤1. High PDZK1 protein expression was classified as tumours with a staining intensity >2, and low expression classified as tumours with a staining intensity ≤2. PBK staining was classified using the ID scoring method (percentage of cells stained multiplied by intensity score), where the threshold for high PBK protein staining was >48. On the basis of this analysis, 309 evaluable tumours (64.5%) were classified as expressing high levels of ANLN and 170 (35.5%) expressing low levels of ANLN; 43 tumours (15.4%) were classified as expressing high levels of PDZK1 versus 237 (84.6%) expressing low levels of PDZK1, and 105 (36.0%) expressing high levels of PBK with 187 (64.0%) expressing low levels of PBK.

### Correlation of ANLN, PDZK1 and PBK protein expression with clinicopathological parameters

On the basis of the IHC thresholds for ANLN, PDZK1 and PBK expression detailed above, we investigated the associations between individual protein expression and a variety of well-defined clinicopathological variables in the TMA cohort (Additional file 1: Table S3). ANLN expression correlated positively with tumour size (*p* = 0.006), high tumour grade (*p <* 0.001), Her2 status (*p* < 0.001), Ki67 status (*p* < 0.001) and invasive ductal carcinomas (IDC) (*p* < 0.001), while correlating negatively with age at diagnosis (p = 0.019), ER status (*p* < 0.001) and PR status (*p* = 0.049). PBK expression correlated positively with high grade tumours (*p* < 0.001) and Ki67 status (*p* < 0.001). PDZK1 expression correlated positively with low grade tumours (*p* = 0.010). There was a significant correlation between ANLN and PBK expression (Pearson’s R = 0.206, *p* < 0.001, n = 283), yet there was no correlation between ANLN and PDZK1 (*p* = 0.410), and PBK and PDZK1 (*p* = 0.543).

### Single marker analysis of ANLN, PDZK1 and PBK protein expression associated with patient survival

**Figure 2:**
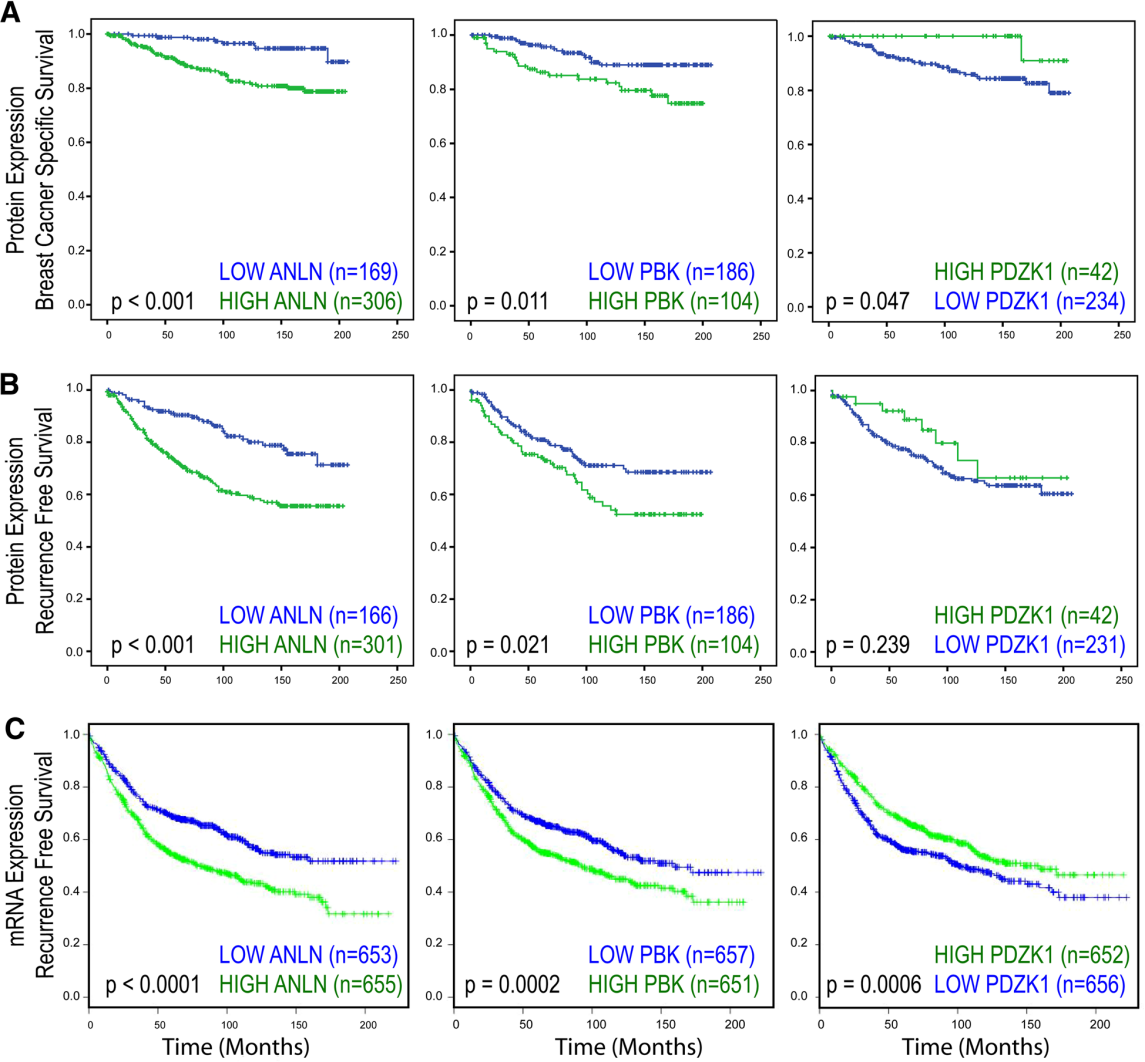
**Prognostic role of ANLN, PBK and PDZK1 at the protein and mRNA level in breast cancer. A**: Kaplan-Meier curves demonstrating high expression of PBK and ANLN protein and low expression of PDZK1 protein associated with reduced BCSS. **B**: Kaplan-Meier curves demonstrating high expression of PBK and ANLN protein and low expression of PDZK1 protein associated with reduced RFS. **C**: Meta-analysis of publicly available transcriptomic data demonstrating high expression of the ANLN and PBK mRNA and low expression of PDZK1 mRNA associated with reduced RFS. P-value represents log-rank test.

The relationship between differential expression of ANLN, PDZK1 and PBK and outcome was subsequently examined. Kaplan-Meier analysis demonstrated that increased PDZK1 protein expression was associated with an improved BCSS (*p* = 0.047), with high levels of ANLN and PBK protein expression being associated with reduced BCSS (ANLN: *p* < 0.001; PBK: *p* = 0.011) (Figure 2A). Univariate Cox regression analysis showed that high ANLN protein expression (HR = 3.91; 95% CI = 1.85-8.29; *p* < 0.001) and high PBK protein expression (HR = 2.33; 95% CI = 1.19-4.55; *p* = 0.013) were associated with reduced BCSS, while differential PDZK1 protein expression (HR = 0.17; 95% CI = 0.02-1.24; *p* = 0.080) was not associated with prolonged BCSS. Both ANLN and PBK were significant independent predictors of BCSS when adjusted for other well-established variables, using multivariate Cox regression analysis (see Additional file 1: Table S4).

The relationship between ANLN, PBK and PDZK1 protein and RFS was examined. Kaplan-Meier analysis showed that high levels of ANLN and PBK protein expression being associated with reduced RFS (ANLN: *p* < 0.001; PBK: *p* = 0.021) (Figure 2B). PDZK1 protein expression was not associated with RFS (p = 0.239). To compare the prognostic impact of ANLN with established factors, Cox regression analysis was performed. Univariate Cox regression analysis confirmed high ANLN expression (HR = 2.41; 95% CI = 1.61-3.62; *p* < 0.001) and high PBK expression were associated with reduced RFS (HR = 1.64; 95% CI = 1.07-3.62; *p* = 0.023). High PDZK1 expression was not associated with prolonged RFS (HR = 0.65; 95% CI = 0.31-1.35; *p* = 0.243). In the multivariate Cox proportional hazards model, ANLN was a significant independent predictor of reduced RFS (HR = 2.14; 95% CI = 1.00-4.58; *p* = 0.038). However, multivariate Cox regression analysis demonstrated that that PBK and PDZK1 protein expression were not independent predictors of RFS (Additional file 1: Table S5).

### mRNA expression levels of ANLN, PDZK1 and PBK in a meta-analysis of publicly available breast cancer transcriptomic datasets

In order to validate these results in a larger number at patients, we performed a meta-analysis of ANLN, PDZK1 and PBK expression from independent transcriptomic datasets, previously described in detail (Additional file 1: Table S2) [36-43]. Using median mRNA expression levels as a cut-off, this meta-analysis displayed high concordance with protein expression data, whereby high expression of ANLN mRNA (*p* < 0.0001), high expression of PBK mRNA (*p* = 0.0002) and low expression of PDZK1 mRNA (*p* = 0.0006) were associated with decreased RFS (Figure 2C). This further confirms the role of ANLN and PBK as poor prognostic markers and PDZK1 as a good prognostic marker.

**Figure 3:**
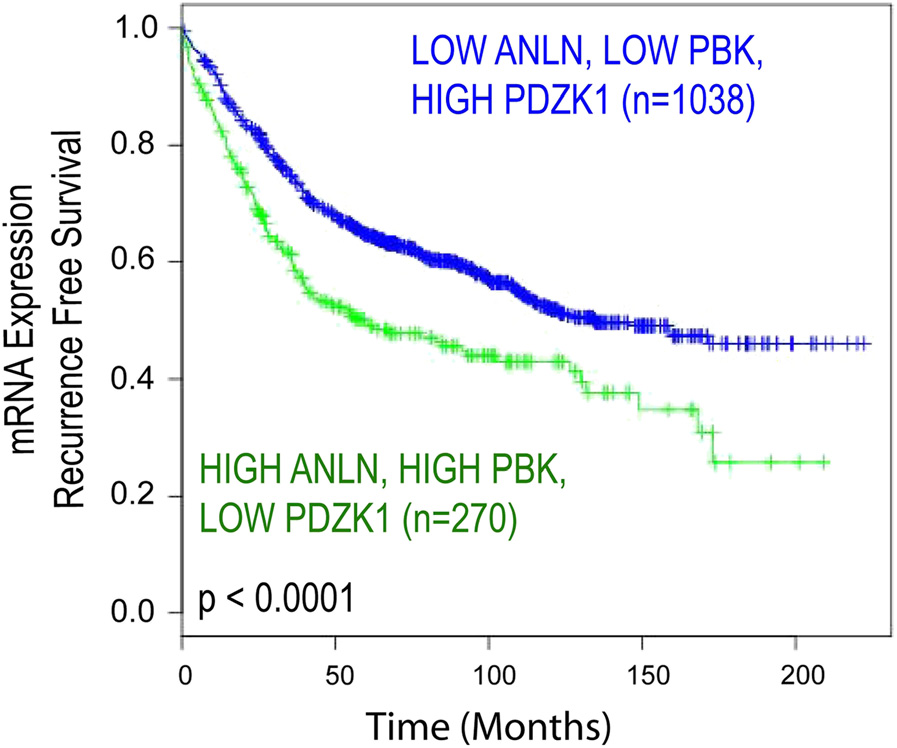
**Transcriptomic screen identifies three markers as a prognostic panel in breast cancer.** Our three-marker model is associated with RFS at mRNA level using a meta-analysis of 10 independent transcriptomic datasets.

By combining these markers into a prognostic signature, we could test the strength of the panel depending on the relative expression of each marker. Patients with the poor prognostic signature (i.e. high expression of ANLN mRNA, high expression of PBK mRNA and low expression of PDZK1 mRNA) had reduced RFS (p < 0.0001, n = 1,308) (Figure 3). Using Multivariate cox regression analysis and adjusting for known clinical parameters, these observations remained independent of lymph node status, tumour grade and ER status (HR = 1.49, 95% CI = 1.08-2.05, *p* = 0.018, n = 699).

### Correlation of 3-protein prognostic panel with clinicopathological parameters and patient survival

Based on the above results, we combined ANLN, PDZK1 and PBK into a 3-protein prognostic model. A scoring technique was devised attributing a score of +1 to each poor prognostic marker exhibited on immunostaining of individual tumour cores (i.e., high ANLN, high PBK and low PDZK1) (Figure 1C). The summed scores for each patient ranged from 0–3 (good → poor prognosis) and, to establish a prognostic model, was dichotomised into a good prognostic set, ‘Signature A’ (incorporating scores 0 and 1), and a poor prognostic set, ‘Signature B’ (incorporating scores 2 and 3).

Two hundred and fifty-two patients of the original cohort had a score for all 3 candidate biomarkers. Associations of the established panel scores (0–3) to known clinicopathological parameters were assessed (Table 1). The panel score was found to correlate with high tumour grade (*p* < 0.001), positive nodal status (*p* = 0.029), ER-negativity (*p* = 0.006), Her2-positivity (*p* = 0.036) and high Ki67 status (*p* < 0.001) status (Table 1).

**Table 1:** Association of panel score with clinicopathological parameters in the consecutive cohort

	**Panel score**				
	**0**	**1**	**2**	**3**	***p*-value**
**Variables**	**(n = 9)**	**(n = 77)**	**(n = 105)**	**(n = 61)**	
**Mean Age**					0.765
≤50	1 (11.1)	11 (14.3)	15 (14.3)	12 (19.7)	
>50	8 (88.9)	66 (85.7)	90 (85.7)	49 (80.3)	
**Tumour Size**					0.475
≤2cm	6 (66.7)	54 (70.1)	66 (62.9)	35 (57.4)	
>2cm	3 (33.3)	23 (29.9)	39 (37.1)	26 (42.6)	
**Histological type**					0.378*
Indeterminate	0 (0.0)	8 (10.4)	5 (4.8)	7 (11.5)	
Ductal	6 (66.7)	47 (61.0)	75 (71.4)	46 (75.4)	
Lobular	2 (22.2)	14 (18.2)	12 (11.4)	4 (6.6)	
Tubular	1 (11.1)	5 (6.5)	7 (6.7)	1 (1.6)	
Medullary	0 (0.0)	0 (0.0)	4 (3.8)	2 (3.3)	
Mucinous	0 (0.0)	3 (3.9)	2 (1.9)	1 (1.6)	
**Tumour Grade**					<0.001*
I	4 (44.4)	25 (32.9)	23 (21.9)	4 (6.6)	
II	5 (55.6)	42 (55.3)	38 (36.2)	19 (31.1)	
III	0 (0.0)	9 (11.8)	44 (41.9)	38 (62.3)	
**Nodal status**					0.029
N0	4 (66.6)	45 (68.2)	49 (51.0)	41 (73.2)	
N1+	2 (33.3)	21 (31.8)	47 (49.0)	15 (26.8)	
Unknown	3	11	9	5	
**ER status**					0.006
ER Negative	0 (0.0)	4 (5.3)	21 (20.6)	14 (23.7)	
ER Positive	8 (100)	72 (94.7)	81 (79.4)	45 (76.3)	
Unknown	1	1	3	2	
**PR status**					0.061
PR Negative	2 (28.6)	16 (26.2)	32 (37.2)	25 (51.0)	
PR Positive	5 (71.4)	45 (73.8)	54 (62.8)	24 (49.0)	
Unknown	2	16	19	12	
**Her2 status**					0.036
0 - 2 +	6 (85.7)	69 (97.2)	88 (87.1)	48 (81.4)	
3+	1 (14.3)	2 (2.8)	13 (12.9)	11 (18.6)	
Unknown	2	6	4	2	
**Ki67 status**					<0.001
0 - 10%	5 (62.5)	48 (62.3)	33 (32.7)	7 (11.7)	
11 - 100%	3 (37.5)	29 (37.7)	68 (67.3)	53 (88.3)	
Unknown	1	0	4	1	

^*^Linear-by-linear χ^2^ analysis; Others by Fisher’s Exact test.

**Figure 4:**
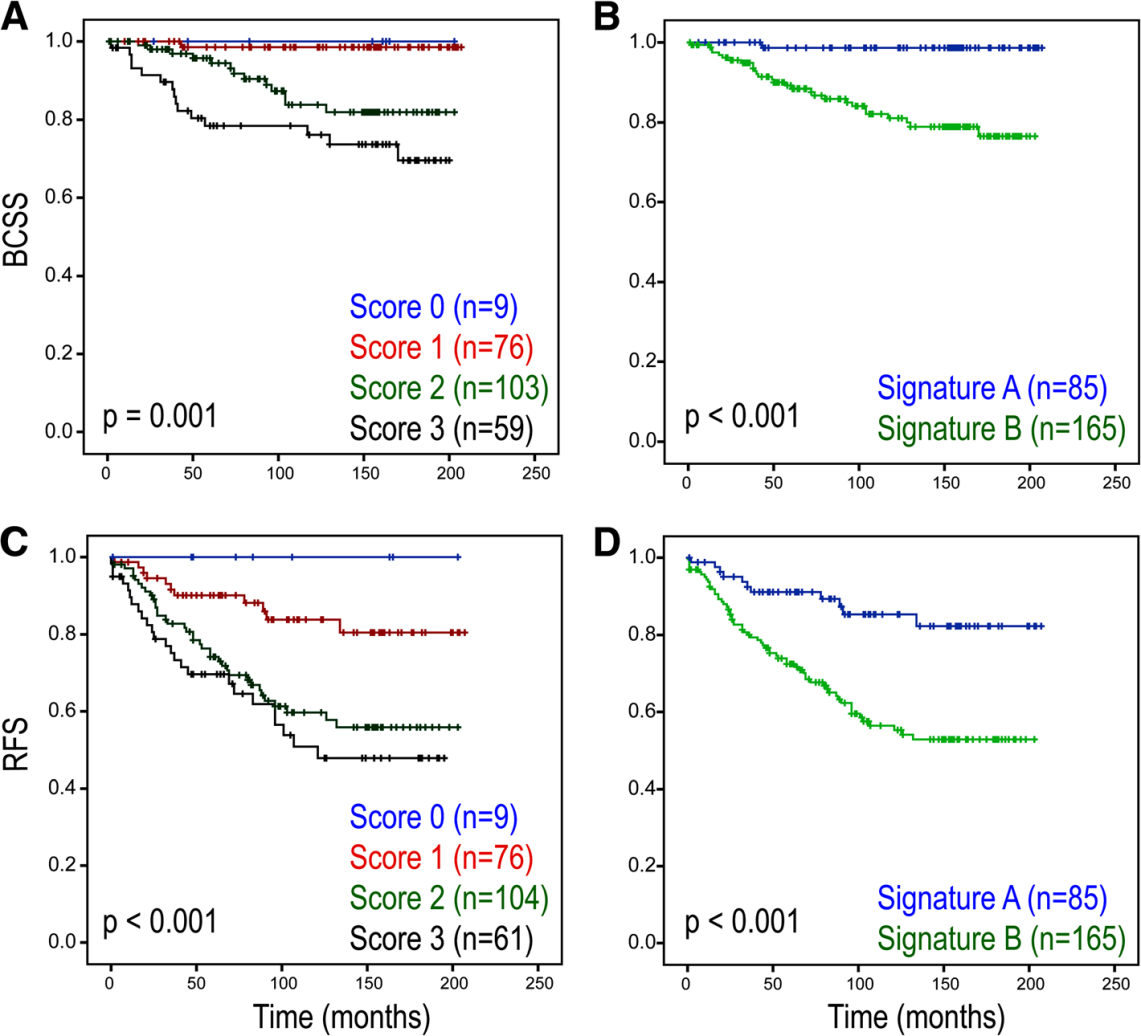
**Novel 3-protein panel as a prognostic model in breast cancer.** Kaplan-Meier curves demonstrating that the three-protein panel is associated with reduced RFS and BCSS; **A**: Individual scores and BCSS, **B**: Dichotimised panel and BCSS, **C**: Individual scores and RFS, **D**: Dichotimised panel and RFS.

When separated into 0, 1, 2 and 3 scores, a higher panel score was significantly associated with poorer BCSS and RFS (Figure 4A and 4C). The dichotomised 3 biomarker panel was significantly predictive of BCSS (*p* < 0.001) (Figure 4B) and RFS (*p* < 0.001) (Figure 4D). To compare the prognostic impact of the panel score with established factors, Cox regression analysis was performed. Univariate Cox regression analysis demonstrated that high panel scores (2 and 3) were significantly associated with reduced BCSS (HR = 16.36; 95% CI = 2.23-120.30; *p* = 0.006) and reduced RFS (HR = 3.33; 95% CI = 1.75-6.31; *p* < 0.001) (summarised in Table 2; all variables listed in Additional file 1: Table S6 and Additional file 1: Table S7). However, multivariate Cox regression demonstrated that the dichotimised 3-panel score was not a significant predictor of either BCSS (HR = 6.38; 95% CI = 0.79-51.26, *p* = 0.082) or RFS (HR = 1.46; 95% CI = 0.66-3.19, *p* = 0.348), when adjusted for other well-established variables, namely tumour grade, tumour size, age at diagnosis, ER, PR, Her2, Ki67 and nodal status. It must be noted when all variables except for PR status are adjusted for, the 3-panel score becomes an independent predictor of BCSS (HR = 11.66; 95% CI = 0.1.50-90.68, *p* = 0.019).

**Table 2:** Cox univariate and multivariate analysis of RFS and BCSS in the consecutive cohort

	** BCSS**		** RFS**	
	**HR (95% CI)**	***p***	**HR (95% CI)**	***p***
All patients (n = 252)				
3 marker panel	Univariate		Univariate	
Signature A	1.00		1.00	
Signature B	16.36 (2.23 - 120.30)	0.006	3.33 (1.75 – 6.31)	<0.001
3 marker panel	Multivariate*		Multivariate*	
Signature A	1.00		1.00	
Signature B	6.38 (0.79 – 51.26)	0.082	1.46 (0.66 – 3.19)	0.348

* Multivariate analysis included adjustment for tumour size (continuous), tumour grade, age at diagnosis (continuous), nodal, ER, PR, Her2 and Ki67 status.

## Discussion

Gene expression profiling has successfully yielded new insights into the biologic diversity of breast cancer identifying several distinct molecular subtypes (such as luminal A, luminal B, basal and Her2) differing markedly in prognosis and in the repertoire of therapeutic targets they express [4,5,50]. Importantly, these intrinsic subtypes play a key role in prediction of disease recurrence, treatment response, and the provision of new insights into oncogenic pathways and metastatic progression [51]. It is striking that, in the face of what is considered a heterogeneous tumour, molecular signatures of tumour subtypes consistently emerge across independent cohorts with diverse genetic and environmental backgrounds [52-54]. This reproducibility is a crucial primary descriptor of disease phenotype in the early detection of disease, lending key prognostic and predictive information.

Antibody-based proteomics occupies a pivotal space within the cancer biomarker discovery and validation pipeline, facilitating the high-throughput evaluation of candidate markers [17]. In this context, IHC-based high-throughput technology has been demonstrated as an effective platform for identification of protein surrogates of these intrinsic breast cancer subtypes by various groups [23,53]. For example, a panel of 5 proteins detected by immunohistochemistry was shown to be prognostic for ER-positive breast cancer [8]. The use of validated IHC surrogates should provide more clinically applicable assays in the future, due to ease of accessibility, low technical demand, cost-effectiveness and applicability to FFPE tissue. Despite these advances, the development of IHC-based assays has been globally impaired by the limited availability of high quality antibodies and lack of rigorous validation of emerging biomarkers. However, the development of comprehensive antibody resources and streamlining of reporting standards, promises to help overcome these obstacles [31,55].

In this study, we sought to determine whether insights from gene expression studies relating to breast cancer progression could be translated into a robust prognostic protein model using a discrete set of IHC markers. This proof-of-concept strategy generated a prognostic panel using high-throughput biomarker screening in combination with a devised panel scoring technique. We confirmed that a high panel score was significantly associated with reduced RFS (*p* < 0.0001; n = 1,038), using a meta-analysis of publicly available breast cancer transcriptomic datasets. The panel was an independent prognostic marker using multivariate Cox regression analysis (*p* = 0.018, HR = 1.49, 95% CI = 1.080-2.054, n = 699). This strategy revealed a novel 3-marker prognostic model significantly predictive of RFS based on ANLN, PDZK1 and PBK expression patterns.

Next, we validated this signature on a protein-based platform using TMA technology. The 3-protein panel score correlated with known pathological prognostic variables, including tumour grade and lymph node status, ER, Her2 and Ki67 status. Univariate Cox regression analysis of RFS demonstrated that high panel scores, indicative of poor prognosis, were significantly associated with reduced RFS. However, multivariate analysis demonstrated that the 3-marker panel score was not a significant predictor of either BCSS (HR = 6.38; 95% CI = 0.79-51.26, *p* = 0.082) or RFS (HR = 1.46; 95% CI = 0.66-3.19, *p* = 0.348), when adjusted for other well-established variables. We noted that the 3-panel score becomes an independent predictor of BCSS (HR = 11.66; 95% CI = 0.1.50-90.68, *p* = 0.019), when all variables except for PR status are adjusted for. This may be due to marginal associations of our individual markers with these variables (e.g. PDZK1 and ER status: *p* = 0.041; PDZK1 and PR status: *p* = 0.074). Since both PDZK1 and PR are surrogate markers for ER activity, we note that the strength of this panel may be skewed by the presence of PDZK1 protein in the panel. Thus, we hypothesise that additional or alternate biochemical markers, interrogated using this high-throughput platform, may further augment the prognostic accuracy of this algorithm to a point that may allow implementation into routine clinical practice.

Interestingly, the 3 proteins that comprise this panel model are associated with distinct pathways in cancer biology. ANLN, initially characterised as a human homologue of anillin, a *Drosophila* actin-binding protein, is essential for the organisation of actin cables in the cleavage furrow, and plays a key role in cytokinesis and cell cycle progression [56-59]. ANLN has been demonstrated as a marker of poor prognosis, relating to aggressive cancer phenotypes [60]. In breast cancer, a transcriptomic study of DCIS to IDC breast cancer progression identified ANLN up-regulation in invasive tumour specimens relative to the pre-invasive phenotype [26]. Our study confirms the role of ANLN as a marker of poor prognosis, at the protein level, in an independent breast cancer cohort. PBK phosphorylates p38MAPK during mitosis, is considered a marker for cellular proliferation and is also implicated in DNA damaging sensing and repair [61,62]. PBK is associated with poorer prognosis in lung cancer [63], is up-regulated in IDC relative to DCIS at the transcriptomic level [26], and may be a promising molecular target for treatment of breast cancer [64]. Our findings further support the role of PBK as a marker of poor prognosis in breast cancer, with expression of PBK also associated with the histological markers of proliferation, Ki67 and tumour grade. PDZK1 is a known estrogen response gene in breast cancer, with proposed roles in signal transduction, cell polarity and ion exchange gating [65,66]. An in-house statistical re-analysis of the genes assessed by van’t Veer and colleagues in the development the 70-gene prognostic signature identified PDZK1 as a marker of good prognosis in breast cancer [24], which we confirmed at the protein level in this study. The present study successfully validates these gene expression findings at the mRNA level, and also translates them at the protein level.

However, further studies are warranted at the *in vitro* and *in vivo* level, to help further interrogate the functional background of each of these markers in breast cancer progression. It will be necessary to further validate these findings with additional independent cohorts of samples to meet accepted international validation guidelines [55]. Although the literature is conflicting with regard to the best way to incorporate histopathology, IHC phenotypes, and gene expression data into an accurate classification system, our findings further support the key role of IHC prognostic models for current breast cancer management.

## Conclusions

We have developed a comprehensive biomarker development pathway, extending from discovery through to validation on TMAs, that can yield novel multi-protein panel signatures for use as a prognostic determinant in breast cancer. Such developments represent an important translational gateway into the era of individualised medicine for patients with newly diagnosed breast cancer.

## Abbreviations

ANLN: Anillin; BCA: Bicinchoninic acid; BCSS: Breast cancer-specific survival; BGA: Between group analysis; AUC: Area under the curve; DAB: Diaminobenzidine; DCIS: Ductal carcinoma *in situ*; ER: Estrogen receptor; FFPE: Formalin-fixed paraffin-embedded; Her2: Human epidermal growth factor receptor 2; His6ABP: Hexa-histidine albumin binding protein; HPA: Human protein atlas; HR: Hazard ratio; HRP: Horseradish peroxidase; ID: Intensity distribution; IDC: Infiltrating ductal carcinoma; IHC: Immunohistochemistry; p38MAPK: p38 mitogen-activated protein kinase; PBK: PDZ-binding kinase; PBS-T: Phosphate-buffered saline with 0.1% Tween 20; PDZK1: PDZ-Domain Containing 1; PR: Progesterone receptor; PrEST: Protein epitope signature tag; RIPA: Radioimmunoprecipitation assay buffer; ROC: Receiver operator curve; RFS: Recurrence-free survival; SDS-PAGE: Sodium dodecyl sulfate polyacrylamide gel electrophoresis; TBS-T: Tris-buffered saline with 0.1% Tween 20; TMA: Tissue microarray.

## Competing interests

The authors (Mathias Uhlèn, Fredrik Pontèn, Karin Jirström) currently hold a patent for the use of ANLN protein as an endocrine treatment predictive factor in breast cancer (Patent US20110269797).

## Authors’ contributions

KJ coordinated the collection of patient tissue and constructed the tissue microarray. FP and MU managed the production of all antibodies. CK, SP and RTD carried out the Western blotting, cell pellet arrays and immunohistochemical analysis. POL, SM, DJB and ER performed the statistical analysis. POL and RTD helped to draft the manuscript. MJD, RZ, AHMc, MRK, KJ and WMG provided critical reading and revision of the manuscript. WMG and KJ conceived of the study, and participated in its design and coordination and helped to draft the manuscript. All authors have read and approved the final manuscript.

## Pre-publication history

The pre-publication history for this paper can be accessed here:

http://www.biomedcentral.com/1471-2407/13/175/prepub

## Supplementary Material

Additional file 1 Supplementary tables. Click here for file

Additional file 2 Supplementary figures. Click here for file
