# Supplementary material for: Systematic antibody generation and validation via tissue microarray technology leading to identification of a novel protein prognostic panel in breast cancer
Source: BMC Cancer. 2013 Apr 2;13:175. doi: 10.1186/1471-2407-13-175 (PMC3668187; doi:10.1186/1471-2407-13-175)
Supplement: Additional file 1 — Supplementary tables. [file 1471-2407-13-175-S1.pdf]

Supplementary Tables

Additional file 1

Supplementary Table 1: List of fifty-six protein targets submitted to the SHPR for target-specific antibody generation. From this submitted list, eighteen antibodies were released for extended analysis.

Ensembl Gene ID	Ensembl Transcript ID	External Gene ID	Gene List	Antibodies Released
ENSG00000124935.2	ENST00000244926.2	SCGB1D2	*BGA re-analysis of top 5000 genes associated with prognosis*<br>Source: Van't Veer et al (Nature 2002)<br>Top 15 genes: Upregulation associated with Good Prognosis	
ENSG00000164128.1	ENST00000296533.1	NPY1R		
ENSG00000110484.1	ENST00000227918.1	SCGB2A2		
ENSG00000175356.2	ENST00000309263.2	SCUBE2		Yes
ENSG00000145824.4	ENST00000337225.2	CXCL14		
ENSG00000174827.4	ENST00000339729.2	PDZK1		Yes
ENSG00000196136.1	ENST00000261981.3	SERPINA3		
ENSG00000144891.7	ENST00000349243.1	AGTR1		
ENSG00000160182.1	ENST00000291527.1	TFF1		
ENSG00000160180.6	ENST00000291525.6	TFF3		
ENSG00000118513.7	ENST00000341545.2	MYB		
ENSG00000124479.3	ENST00000244349.1	NDP		
ENSG00000178568.4	ENST00000342788.1	ERBB4		Yes
ENSG00000120875.1	ENST00000240100.1	DUSP4		
ENSG00000116176.4	ENST00000234798.2	TPSG1		
ENSG00000185686.5	ENST00000358617.1	PRAME	*BGA re-analysis of top 100 genes associated with prognosis*<br>Source: Van't Veer et al (Nature 2002)<br>Top 16 genes: Upregulation associated with Poor Prognosis	
ENSG00000167614.3	ENST00000301194.2	TTYH1		
ENSG00000143546.1	ENST00000271846.1	S100A8		
ENSG00000163220.3	ENST00000295382.2	S100A9		
ENSG00000007062.2	ENST00000265014.2	PROM1		Yes
ENSG00000127324.2	ENST00000247829.2	TM4SF3		
ENSG00000109255.1	ENST00000264218.1	NMU		
ENSG00000136943.2	ENST00000259470.2	CTSL2		
ENSG00000183036.2	ENST00000328619.2	PCP4		Yes
ENSG00000134760.2	ENST00000257192.2	DSG1		
ENSG00000169679.2	ENST00000302759.2	BUB1		
ENSG00000107159.2	ENST00000291153.2	CA9		
ENSG00000130829.7	ENST00000361486.1	DUSP9		

ENSG00000117650.2	ENST00000261456.2	NEK2		
ENSG00000164109.3	ENST00000296509.2	MAD2L1		
ENSG00000126977.1	ENST00000247413.1	MAGEA1		
ENSG00000011426.2	ENST00000265748.1	ANLN	*Genes associated with DCIS to invasive ductal carcinoma transition*	Yes
ENSG00000013810.6	ENST00000313288.2	TACC3	Source: Ma et al (PNAS 2003)	Yes
ENSG00000076003.1	ENST00000264156.1	MCM6	25 Genes: upregulated in IDC Vs DCIS	
ENSG00000096238.2	ENST00000211475.2	CLIC1		Yes
ENSG00000100526.6	ENST00000335183.3	CDKN3		
ENSG00000103187.2	ENST00000262428.2	COTL1		Yes
ENSG00000111665.2	ENST00000229265.1	CDCA3		Yes
ENSG00000115310.7	ENST00000357376.1	RTN4		
ENSG00000123975.1	ENST00000243756.1	CKS2		
ENSG00000131747.5	ENST00000357601.1	TOP2A		Yes
ENSG00000135476.2	ENST00000257934.2	ESPL1		
ENSG00000143416.7	ENST00000290533.6	SELENBP1		Yes
ENSG00000145020.3	ENST00000273588.2	AMT		Yes
ENSG00000158402.7	ENST00000337493.1	CDC25C		
ENSG00000162896.2	ENST00000356495.1	PIGR		Yes
ENSG00000168078.1	ENST00000301905.1	PBK		Yes
ENSG00000168393.5	ENST00000305784.1	DTYMK		
ENSG00000171848.2	ENST00000304567.2	RRM2		
ENSG00000173540.3	ENST00000308388.2	GMPPB		Yes
ENSG00000175063.7	ENST00000343198.1	UBE2C		Yes
ENSG00000176619.2	ENST00000325327.2	LMNB2		
ENSG00000182054.1	ENST00000330062.1	IDH2		Yes
ENSG00000188042.2	ENST00000339728.2	ARL7		
ENSG00000188547.2	ENST00000341784.1	DTYMK		
ENSG00000143320.1	ENST00000271558.1	CRABP2		

Supplementary Table 2: List of ten publicly available transcriptomic datasets included in a meta-analysis to evaluate the individual prognostic significance of candidate proteins (ANLN, PBK, PDZK1) at the transcriptomic level.

Reference	GEO Accession Number	Availability	Sample Number	Platform Type
Calabro et al., 2009 [4]	GSE10510	Raw data avaiable	152	DKFZ Division of Molecular Genome Analysis Human Operon 4.0 oligo Array 35k
Chang et al., 2005 [5]	NA	Processed only	295	Agilent
Desmedt et al., 2009 [6]	GSE16391	Raw CEL files	48	Affymetrix U133 Plus 2.0
Hu et al., 2006 [7]	GSE1992	Processed only	99	Agilent
Kok et al., 2009 [8]	NA	Processed only	109	Agilent 44K oligo array
Loi et al., 2008 [9]	GSE9195	Raw CEL files	77	Affymetrix U133 Plus 2.0
Loi et al., 2008 [9]	GSE6532	Raw CEL files	87	Affymetrix U133A/B and plus2
Sabatier et al., 2010 [10]	GSE21653	Raw CEL files	266	Affymetrix U133 Plus 2.0
Sabatier et al., 2010 [10]	GSE17907	Raw CEL files	51	Affymetrix U133 Plus 2.0
Chanrion et al., 2008 [11]	GSE9893	Raw data available	155	MLRG Human 21K V12.0

Supplementary Table S3: Association of PDZK1, PBK and ANLN expression with clinicopathological parameters in the consecutive cohort

	PDZK1 Expression			PBK Expression			ANLN Expression		
Variable	Low PDZK1 (n = 237)	High PDZK1 (n = 43)	*p*-value	Low PBK (n=187)	High PBK (n=105)	*p*-value	Low ANLN (n = 170)	High ANLN (n = 309)	*p*-value
**Mean Age**			1.000			0.308			**0.019**
<50 years#	37 (15.6)	7 (16.3)		25 (13.4)	19 (18.1)		18 (10.6)	58 (18.8)	
>50 years#	200 (84.4)	36 (83.7)		162 (86.6)	86 (81.9)		152 (89.4)	251 (81.2)	
**Tumour Size**			0.866			0.523			**0.006**
1 - 19mm	152 (64.1)	27 (62.8)		121 (64.7)	64 (61.0)		119 (70.0)	177 (57.3)	
> 20mm	85 (35.9)	16 (37.2)		66 (35.3)	41 (39.0)		51 (30.0)	132 (42.7)	
**Histological subtype**			0.562*			0.124*			**<0.001***
Indeterminate	17 (7.2)	4 (9.3)		12 (6.4)	11 (10.5)		11 (6.5)	22 (7.1)	
IDC	158 (66.7)	33 (76.7)		122 (65.2)	78 (74.3)		95 (55.9)	224 (72.5)	
ILC	35 (14.8)	4 (9.3)		27 (14.4)	10 (9.5)		40 (23.5)	29 (9.4)	
Tubular	13 (5.5)	1 (2.3)		16 (8.6)	2 (1.9)		15 (8.8)	14 (4.5)	
Medullary	9 (3.8)	0 (0.0)		5 (2.7)	2 (1.9)		0 (0.0)	13 (4.2)	
Mucinous	5 (2.1)	1 (2.3)		5 (2.7)	2 (1.9)		9 (5.3)	7 (2.3)	
**Tumour Grade**			**0.01***			**<0.001***			**<0.001***
I	49 (20.7)	11 (26.2)		56 (30.1)	14 (13.3)		66 (38.8)	52 (16.9)	
II	97 (40.9)	25 (59.5)		80 (43.0)	40 (38.1)		84 (49.4)	117 (38.0)	
III	91 (38.4)	6 (14.3)		50 (26.9)	51 (48.6)		20 (11.8)	139 (45.1)	
Unknown	0	1		1	0		0	1	
**Nodal status**			0.261			0.283			0.058
N0	131 (61.5)	27 (71.1)		99 (60.0)	64 (66.7)		102 (68.5)	165 (59.1)	
N1+	82 (38.5)	11 (28.9)		66 (40.0)	32 (33.3)		47 (31.5)	114 (40.9)	
Unknown	24	5		22	9		21	30	
**ER status**			**0.041**			0.251			**<0.001**
ER Negative	41 (17.8)	2 (5.0)		26 (14.4)	20 (19.6)		6 (3.8)	60 (20.4)	

ER Positive	189 (82.2)	38 (95.0)		155 (85.6)	82 (80.4)		154 (96.2)	234 (79.6)	
Unknown	7	3		6	3		10	15	
**PR status**			0.074			0.078			**0.044**
PR Negative	75 (39.3)	7 (22.6)		51 (34.2)	39 (45.9)		36 (30.5)	98 (41.5)	
PR Positive	116 (60.7)	24 (77.4)		98 (65.8)	46 (54.1)		82 (69.5)	138 (58.5)	
Unknown	46	12		38	20		52	73	
**HER2**			0.273			0.189			**<0.001**
0 - 2 +	193 (87.3)	36 (94.7)		156 (91.2)	87 (86.1)		152 (97.4)	259 (86.3)	
3+	28 (12.7)	2 (5.3)		15 (8.8)	14 (13.9)		4 (2.6)	41 (13.7)	
Unknown	16	5		16	4		14	9	
**Ki67**			0.462			**<0.001**			**<0.001**
0 – 10%	87 (37.8)	18 (43.9)		84 (46.9)	26 (25.0)		110 (67.9)	61 (21.0)	
11 - 100%	143 (62.2)	23 (56.1)		95 (53.1)	78 (75.0)		52 (32.1)	229 (79.0)	
Unknown	7	2		8	1		8	19	

*Linear-by-linear χ^2 analysis; Others by Fisher's Exact test; #years at diagnosis

Supplementary Table S4: Cox regression analysis of individual marker expression in relation to BCSS in the consecutive cohort.

Prognostic Factor	Univariate			Multivariate*		
	**HR**	**95% CI**	***p*-value**	**HR**	**95% CI**	**p-value**
PDZK1 (high *vs.* low, ref)	0.169	0.023 – 1.239	0.080	-	-	0.969
PBK (high *vs.* low, ref)	2.329	1.191 – 4.554	0.013	3.847	1.461 – 10.131	0.006
ANLN (high *vs.* low, ref)	3.914	1.849 – 8.287	<0.001	4.596	1.147 – 18.424	0.031
Tumour Size (continuous)	1.016	1.008 – 1.024	<0.001	1.021	1.003 - 1.040	0.022
Nodal status (pos *vs.* neg, ref)	5.142	2.910 – 9.088	<0.001	5.671	1.823 – 17.645	0.003
Tumour Grade (3 *vs.* 1-2, ref)	4.990	2.892 – 8.610	<0.001	2.788	0.907 – 8.569	0.073
ER status (pos *vs.* neg, ref)	0.516	0.272 – 0.981	0.043	2.422	0.571 – 10.271	0.230
PR status (pos *vs.* neg, ref)	0.274	0.146 - 0.513	<0.001	0.333	0.111 – 1.000	0.050
Ki67 status (>10% vs. ≤10%, ref)	2.977	1.498 – 5.917	0.002	4.747	0.186 – 3.119	0.704
Her2 status (2-3 *vs.* 0-1, ref)	1.090	0.467 - 2.543	0.842	0.761	0.186 – 3.119	0.704
Tumour type (categorical)	0.700	0.496 – 0.989	0.043	-	-	-
Age (continuous)	0.986	0.965 – 1.007	0.184	0.570	0.212 – 1.529	0.264

*Adjusted for all other variables in the table; ref, referent group.

Supplementary Table S5: Cox regression analysis of individual marker expression in relation to RFS in the consecutive cohort.

Prognostic Factor	Univariate			Multivariate*		
	HR	95% CI	*p*-value	HR	95% CI	p-value
PDZK1 (high *vs.* low, ref)	0.647	0.311 – 1.345	0.243	0.713	0.257 – 1.979	0.516
PBK (high *vs.* low, ref)	1.636	1.071 – 3.614	0.023	1.393	0.785 – 2.472	0.257
ANLN (high *vs.* low, ref)	2.409	1.606 – 3.614	<0.001	2.122	0.995 – 4.523	0.051
Tumour Size (continuous)	1.009	1.003 – 1.015	0.003	1.005	0.991 - 1.019	0.505
Nodal status (pos *vs.* neg, ref)	3.368	2.382 – 4.763	<0.001	2.867	1.493 – 5.507	0.002
Tumour Grade (3 *vs.* 1-2, ref)	3.156	2.274 – 4.381	<0.001	2.302	1.176 - 4.505	0.015
ER status (pos *vs.* neg, ref)	0.706	0.460 – 1.082	0.110	1.957	0.765 – 5.005	0.161
PR status (pos *vs.* neg, ref)	0.513	0.351 - 0.749	0.001	0.483	0.246 - 0.949	0.035
Ki67 status (>10% vs. ≤10%, ref)	1.911	1.310 – 2.788	0.001	1.103	0.516 – 2.359	0.801
Her2 status (2-3 *vs.* 0-1, ref)	0.792	0.437 -1.433	0.441	0.542	0.191 – 1.542	0.251
Tumour type (categorical)	0.774	0.639 – 0.938	0.009	-	-	-
Age (continuous)	1.003	0.989 – 1.016	0.700	0.706	0.363 – 1.375	0.307

*Adjusted for all other variables in the table; ref, referent group.

Supplementary Table S6: Cox regression analysis of clinicopathological parameters and panel score in relation to BCSS in the consecutive cohort

Prognostic Factor	Univariate			Multivariate*		
	**HR**	**95% CI**	***p*-value**	**HR**	**95% CI**	**p-value**
3 Panel Score (2-3 *vs.* 0-1, ref)	16.363	2.226 – 120.295	0.006	6.375	0.793 – 51.528	0.082
Tumour Size (continuous)	1.016	1.008 – 1.024	<0.001	0.982	0.948 – 1.017	0.035
Nodal status (pos *vs.* neg, ref)	5.142	2.910 – 9.088	<0.001	2.744	0.987 – 7.624	0.053
Tumour Grade (3 *vs.* 1-2, ref)	4.990	2.892 – 8.610	<0.001	3.061	1.023 – 9.159	0.045
ER status (pos *vs.* neg, ref)	0.516	0.272 – 0.981	0.043	3.635	0.848 – 15.583	0.082
PR status (pos *vs.* neg, ref)	0.274	0.146 - 0.513	<0.001	0.256	0.084 – 0.775	0.016
Ki67 status (>10% vs. ≤10%, ref)	2.977	1.498 – 5.917	0.002	1.603	0.433 – 5.933	0.480
Her2 status (2-3 *vs.* 0-1, ref)	1.090	0.467 - 2.543	0.842	0.799	0.195 – 3.279	0.756
Tumour type (categorical)	0.700	0.496 – 0.989	0.043	-	-	-
Age (continuous)	0.986	0.965 – 1.007	0.184	0.982	0.982 – 1.017	0.308

*Adjusted for all other variables in the table; ref, referent group.

Supplementary Table S7: Cox regression analysis of clinicopathological parameters and panel score in relation to RFS in the consecutive cohort

Prognostic Factor	Univariate			Multivariate*		
	HR	95% CI	*p*-value	HR	95% CI	p-value
3 Panel Score (2-3 *vs.* 0-1, ref)	3.325	1.752 – 6.310	<0.001	1.456	0.665 – 3.190	0.348
Tumour Size (continuous)	1.009	1.003 – 1.015	0.003	1.000	0.978 - 1.023	0.994
Nodal status (pos *vs.* neg, ref)	3.368	2.382 – 4.763	<0.001	2.521	1,346 – 4.722	0.004
Tumour Grade (3 *vs.* 1-2, ref)	3.156	2.274 – 4.381	<0.001	2.326	1.184 - 4.567	0.014
ER status (pos *vs.* neg, ref)	0.706	0.460 – 1.082	0.110	1.849	0.714 – 4.789	0.206
PR status (pos *vs.* neg, ref)	0.513	0.351 - 0.749	0.001	0.487	0.252 - 0.940	0.032
Ki67 status (>10% vs. ≤10%, ref)	1.911	1.310 – 2.788	0.001	1.322	0.648 – 2.697	0.443
Her2 status (2-3 *vs.* 0-1, ref)	0.792	0.437 -1.433	0.441	0.534	0.187 – 1.525	0.242
Tumour type (categorical)	0.774	0.639 – 0.938	0.009	-	-	-
Age (continuous)	1.003	0.989 – 1.016	0.700	1.000	0.978 – 1.023	0.994

*Adjusted for all other variables in the table; ref, referent group.
