# Supplementary figures and images for: Systematic antibody generation and validation via tissue microarray technology leading to identification of a novel protein prognostic panel in breast cancer
Source: BMC Cancer. 2013 Apr 2;13:175. doi: 10.1186/1471-2407-13-175 (PMC3668187; doi:10.1186/1471-2407-13-175)

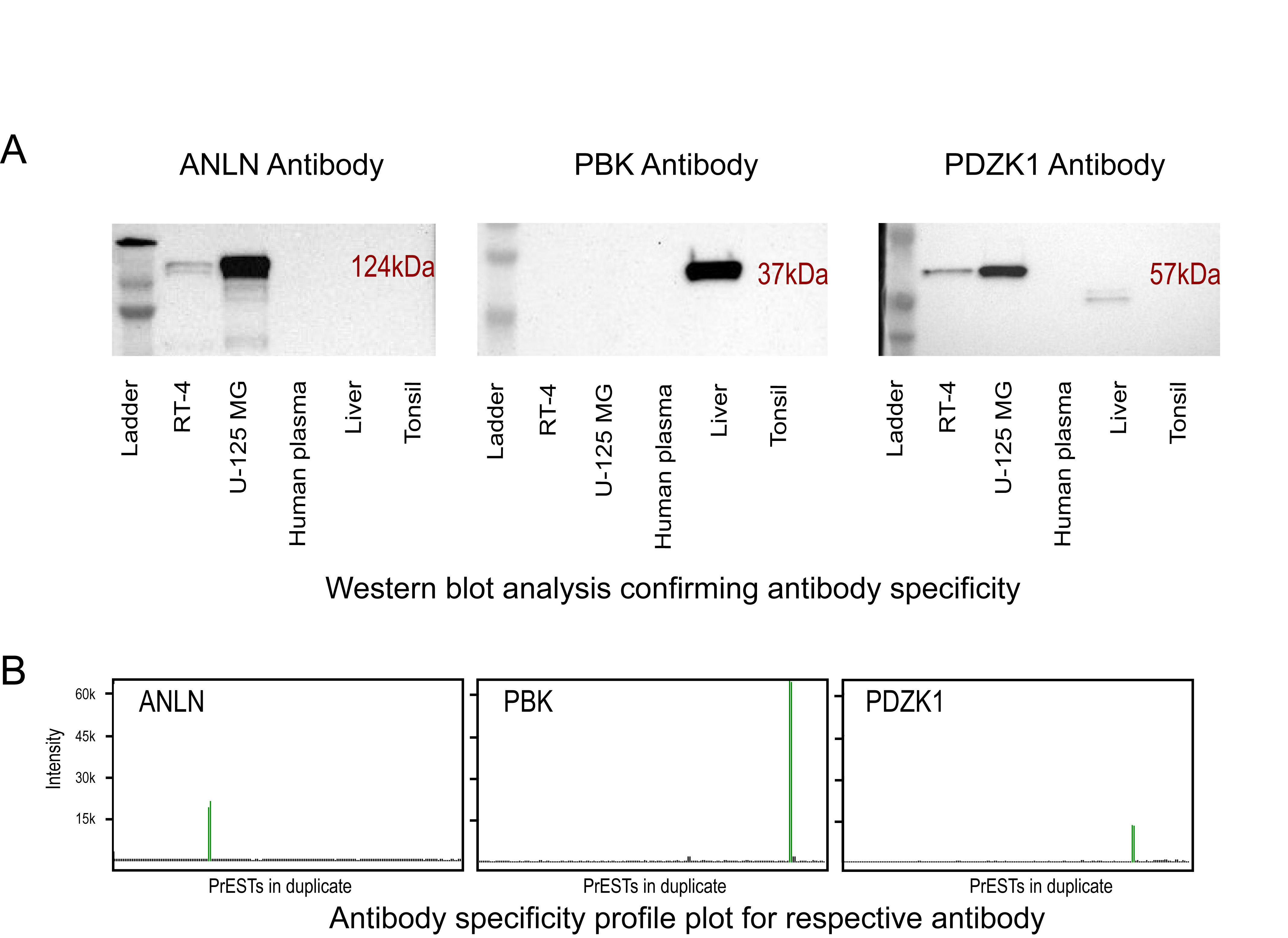

Supplement: Additional file 2 — Supplementary figures. [file 1471-2407-13-175-S2.jpeg]
